# Psychedelics for Brain Injury: A Mini-Review

**DOI:** 10.3389/fneur.2021.685085

**Published:** 2021-07-29

**Authors:** Mia Khan, Gregory T. Carter, Sunil K. Aggarwal, Julie Holland

**Affiliations:** ^1^Washington University School of Medicine in St. Louis, St. Louis, MO, United States; ^2^Department of Physical Medicine & Rehabilitation, St. Luke's Rehabilitation Institute, Spokane, WA, United States; ^3^Advanced Integrative Medical Sciences Institute, Seattle, WA, United States; ^4^Private Practitioner, New York, NY, United States

**Keywords:** psychedelics, neuroinflammation, neuroplasticity, stroke, brain injury, review

## Abstract

**Objective:** Stroke and traumatic brain injury (TBI) are among the leading causes of disability. Even after engaging in rehabilitation, nearly half of patients with severe TBI requiring hospitalization are left with major disability. Despite decades of investigation, pharmacologic treatment of brain injury is still a field in its infancy. Recent clinical trials have begun into the use of psychedelic therapeutics for treatment of brain injury. This brief review aims to summarize the current state of the science's relevance to neurorehabilitation, and may act as a resource for those seeking to understand the precedence for these ongoing clinical trials.

**Methods:** Narrative mini-review of studies published related to psychedelic therapeutics and brain injury.

**Results:** Recent *in vitro, in vivo*, and case report studies suggest psychedelic pharmacotherapies may influence the future of brain injury treatment through modulation of neuroinflammation, hippocampal neurogenesis, neuroplasticity, and brain complexity.

**Conclusions:** Historical data on the safety of some of these substances could serve in effect as phase 0 and phase I studies. Further phase II trials will illuminate how these drugs may treat brain injury, particularly TBI and reperfusion injury from stroke.

## Introduction

Despite millennia of historical use around the world, research into medical uses of psychedelic drugs has been stymied for years by stigma (1). “Classical” psychedelic drugs refer to the most well studied and culturally significant psychedelics, including mescaline, lysergic acid diethylamide (LSD), psilocybin, and dimethyltryptamine (DMT). Though having a wide range of molecular structures and target receptors, psychedelics are unified by their ability to produce marked alterations in sensory perception, consciousness, distortion of time, and perception of reality. Evidence suggests that activation of 5-HT2A receptors (a class of excitatory receptors of serotonin or 5-HT), is the common mechanism for the psychological experience of classical psychedelics, though these compounds are known to act at other receptors (2).

Currently, however, psychedelics are experiencing a scientific renaissance due to advances in research methodology and changes in the regulation of these substances. Trials of psilocybin for disorders of consciousness, and DMT for stroke, are in discussion to begin in coming years (3, 4). *In vitro* and *in vivo* studies suggest psychedelics may influence the future of brain injury treatment in both the acute and chronic phases through a variety of mechanisms including modulation of neuroinflammation, neuroplasticity, hippocampal neurogenesis, and increases in brain complexity.

## Methods

We conducted a literature search of articles relevant to psychedelic therapeutics for brain injury using PubMed. The search terms are available in the Appendix. Bibliographies of the main review papers were also used to detect other relevant articles. Studies were selected with emphasis to their relevance to psychedelics' purported neuroregenerative and neuroprotective potential, rather than their psychotherapeutic properties. A narrative mini-review format was employed with the intention of providing a brief overview for readers seeking to understand the scientific basis for anticipated clinical trials.

## Neuroinflammation

Within the brain, depression, addiction, Alzheimer's, and Parkinson's all appear to be linked to neuroinflammatory states (5–7). There are currently three main classes of anti-inflammatory drugs: non-steroidal anti-inflammatory drugs (NSAIDs), steroids such as prednisone, and biologics which act like sponges to “soak up” inflammatory cytokines (1). Psychedelics may represent a fourth class of anti-inflammatory drug.

Neuroinflammation after stroke is responsible for both infarct expansion as well as remodeling and repair (8, 9). Modulation of this inflammation is currently a target for new therapies. The inflammatory response to ischemic stroke is thought to derive largely from reperfusion injury (10). In general, there are no conventional medical therapies addressing reperfusion injury after stroke, with the exception of edavarone in Japan, which is only modestly effective (11).

After stroke, immune cells invade the injured tissue, interacting with microglia and neurons (8). Modulating this inflammatory response, particularly through tumor necrosis factor (TNF), interleukin (IL)-1, IL-6, and IL-10, may be the next frontier in stroke recovery (8, 12). However, it should be expected that the cytokine response to brain injury has both beneficial and harmful effects to the recovering patient. In contrast to steroids, which cause generalized systemic immunosuppression, psychedelics produce a unique pattern of cytokine expression favoring anti-allergic conditions (13, 14). In other words, psychedelics may target many of the pathologic immune responses without exposing the body to the risks of total immune suppression (e.g., serious infection) or potential side effects of existing biologics (e.g., malignancy and cardiovascular disease). Instead, careful regulation of the inflammatory response, rather than blunt reduction of the response, or “single-target” approaches, is critical to improved outcomes.

Mizuma and Yenari (10) argue that immunomodulatory therapies have previously not shown efficacy in clinical trials because they largely took place before the use of revascularization techniques. Because reperfusion injury is thought to be the inciting event for much of the neuroinflammatory response, they argue that combining revascularization with immunomodulation may hold promise for stroke treatment. Indeed, Jickling et al.'s (12) review of neutrophil modulation as treatment for stroke notes the contrast seen in the efficacy of these treatments in animal models is dependent on whether reperfusion is induced.

Classical psychedelics act principally on the 5-hydroxytryptamine receptors (5-HTRs) to produce their psychological effects, specifically the 5-HT2a receptor (5). These same receptors are well-known to have the potential to regulate inflammation within the central nervous system and peripherally (15). In fact, the 5-HT2a receptor is the most widely expressed serotonin receptor throughout the human body (1). It is present on nearly all tissue and cell types, including all major immune-related cell types (1). However, the highest density of 5-HT2a receptors is found in the brain (1). Though peripheral immunomodulation has been documented with other psychedelics like lysergic acid diethylamide (LSD) (16), 3,4-methylenedioxy-methamphetamine (MDMA) (17), and 2,5-dimethoxy-4-iodoamphetamine (DOI) (13), N,N-Dimethyltryptamine (DMT) has been especially well-studied with regards to its effects on neuroinflammation and reperfusion injury.

DMT is a psychedelic which is endogenously produced by the human brain, particularly the pineal gland, likely in local concentrations comparable to other monoamine neurotransmitters (18). Though it has been known for at least 40 years that DMT is an endogenously produced hallucinogen, its physiologic function remains elusive. The lack of consensus may be due in part to a paradigm in which the scientific community has assumed DMT can only be, or primarily acts as, a hallucinogen, which keeps research focus on its psychologic effects at 5-HTRs, rather than its non-hallucinogenic effects (9). Recent experiments have shown that an additional receptor, S1R, is critical in the immunomodulating response of DMT.

S1R dysfunction is known to be involved in a wide range of neurodegenerative diseases including Alzheimer's disease, Parkinson's disease, Huntington's disease, amyotrophic lateral sclerosis (ALS), and TBI (19–21). S1R agonists produce neuroprotective effects *via* regulation of intracellular calcium, reducing expression of pro-apoptotic genes, and inhibiting anti-apoptotic activity by the Bcl-2 gene (22). Specifically, DMT's action at S1Rs has been shown in murine models to modulate inflammation by reducing IL-1b, IL-6, TNFa, and IL-8, while increasing the secretion of the anti-inflammatory cytokine IL-10, and by inhibiting activation of Th1 (T Helper cell type 1) and Th17 (T Helper cell type 17) subsets (20). In mice, S1R is known to play an important role in the endoplasmic reticulum stress response, including oxidative stress, probably by up-regulating antioxidants, quinine oxidoreductase 1 and superoxide dismutase (SOD) (23). Being present in reactive astrocytes, microglia, and neurons, it has also been shown to regulate neuritic outgrowth, myelination, synaptogenesis, and neuro-regeneration (22, 24). It should be noted that S1R is now known to have multiple endogenous agonists, such as progesterone (25). However, with no clear alternative explanation, it may be reasonable to hypothesize that DMT has some, or all, of these neuroprotective and anti-inflammatory physiologic functions as well. The only clinical trial of a selective S1R agonist for treatment of stroke showed statistically significant functional recovery in *post-hoc* analysis of moderately-to-severely affected patients (26).

DMT has been shown to reduce ischemic brain injury after middle cerebral artery occlusion (MCAO) through S1R dependent activity in murine models (27). Nardai et al. (27) documented a significant reduction of the infarct core volume in rats at 24 h post MCAO with administration of DMT, as well as enhanced functional regeneration of the affected limb at 30 days post MCAO (27). These results are consistent with other *in vitro* studies showing cytoprotective effects of DMT against reperfusion injury (28). Furthermore, similar effects have been documented in human-specific models including human cerebral organoids. Dakic et al. (22) were the first to document that a closely related congener of DMT, 5-MeO-DMT, found in high concentrations of the toxin of *Incilius alvarius* (Colorado River Toad), favorably altered the cerebral proteome with regard to factors involved with plasticity and neuroprotection including long-term potentiation, apoptosis, morphogenesis/maturation of dendritic spines, and T-lymphocyte differentiation, while inhibiting factors involved in neurodegeneration and cell death. The authors note that other dimethyltryptamines may have similar effects, but their results show that the mechanisms may be different and that each deserves careful study. Previous studies in monolayer neuronal cultures did not show the same effects, suggesting a more complex circuitry is required for these effects (22).

## Hippocampal Neurogenesis

TBI and stroke alter hippocampal neurogenesis in murine models (29, 30). Though hippocampal neurogenesis is recognized as an important component of cognitive recovery from TBI and stroke, there is not a direct correlation between increased neurogenesis and recovery. Complicating factors include the nature of the injury, the timing of intervention, how the cells integrate into the hippocampal circuits, and whether the target of intervention is either increased neuronal proliferation or increased survival. While hippocampal neurogenesis after TBI is implicated in improved cognition, relief from depressed mood, and encoding of episodic memory, it is also associated with pro-epileptogenic changes and spatial memory impairment (29, 31, 32).

Though many factors are implicated in hippocampal neurogenesis, one of the most important is 5HTR stimulation (33, 34). Acute administration of psilocybin to mice alters hippocampal neurogenesis in a non-linear fashion (35). Low doses lead to increased neurogenesis while higher doses inhibit it. However, increased neurogenesis has also been seen when high dose psilocybin was administered once-per-week, avoiding the issue of rapid tolerance buildup *via* 5HTR downregulation (33). Targeting hippocampal neurogenesis for treatment of brain injury and other psychiatric and neurologic disorders is an emerging area of research (36).

## Neuroplasticity

Novel interventional approaches hold promise to improve functional outcomes after brain injury by inducing neural plasticity. However, like targeting inflammation and hippocampal neurogenesis, targeting neuroplasticity is a double-edged sword. Ischemia induced plasticity may be responsible for recovery of function, but also drive complications such as epilepsy and memory disturbance. A full discussion on ischemia induced plasticity is available elsewhere (37).

Functional recovery due to post-stroke plasticity is currently most effectively recruited through intensive physical therapy (37). However, both non-invasive techniques for inducing neuroplasticity such as transcranial magnetic stimulation and direct current stimulation, as well as invasive techniques such as deep brain stimulation, show promising results for stroke recovery (37).

The search for pharmacologic agents which stimulate neuroplasticity after brain injury, including amphetamines, dopaminergic, serotonergic, noradrenergic, and cholinergic agents, has thus far been inconclusive. To our knowledge, the effect of psychedelics on neuroplasticity has not been tested in brains subjected to injury such as stroke. However, recent reports demonstrate that psychedelics promote both structural and functional neuroplasticity in non-injured brains. The persistent symptom improvement in psychiatric disorders with administration of psychedelics has been proposed to be driven by this neuroplastic adaptation (2). Ly et al. (38) found that some psychedelics were more efficacious (e.g., MDMA) or more potent (e.g., LSD) than ketamine in promoting plasticity. These results were demonstrated *in vivo* in both non-human vertebrates and invertebrates, suggesting that these mechanisms are evolutionarily conserved.

One hypothesized mechanism for psychedelics' beneficial effect on neuroplasticity in brain injury is *via* Brain-derived neurotrophic factor (BDNF), which is well-known to be implicated in both neuritogenesis and spinogenesis (39). In Ly et al.'s (38) experiments, they found that many psychedelics rivaled administration of pure BDNF in their ability to induce plasticity. At least one randomized control trial has demonstrated increases in serum BDNF in volunteers with administration of a DMT containing tisane, ayahuasca, which persisted for 48 h (40). Importantly, ayahuasca, a traditional South American ceremonial brew, contains not only DMT, but also β-carbolines which act as reversible monoamine oxidase inhibitors (MAOIs) potentiating and prolonging the effects of DMT (40). However, it should be noted that many other non-psychedelic drugs have failed to alter the course of brain injury through induction of BDNF.

However, there is also evidence to suggest that S1R is an important part of the plasticity response to stroke. For example, Ruscher et al. (41) found that delivery of a synthetic S1R agonist enhanced plasticity-mediated recovery of lost sensorimotor function in rats, even when initiation of therapy was started up to 2 days after MCAO, possibly expanding the currently very narrow therapeutic window for acute stroke.

Mirror visual-feedback (MVF) has been shown to enhance key features of neuroplasticity including cross-modal cortical reorganization and learning (42). In patients <12 months post stroke, MVF therapy enhances functional recovery of lower limbs and hands (43, 44). Psilocybin combined with mirror visual-feedback has been shown in a case report to have a dose-dependent reduction in phantom limb associated pain, hypothesized to be due to facilitating MVF's ability to “unlearn” paralysis *via* 5HTR-dependent changes in neuroplasticity (42). Clinical trials for use of psilocybin in phantom limb pain are currently underway (45).

## Increase in Brain Complexity

Disorders of consciousness (DOC) can arise from a variety of brain injuries including trauma, hypoglycemia, anoxia, and stroke. Between 4 and 38% of stroke patients will experience a DOC (46). Though many therapies have been proposed for patients with DOC, including pharmacologic (e.g., amantadine, D-amphetamine, levodopa, modafinil, and zolpidem), invasive and non-invasive stimulation (e.g., transcranial direct current stimulation), benefits for functional recovery are usually modest and current evidence supporting their use is inconsistent (47–50). Other stimulants acting on dopamine including apomorphine may hold promise (51).

Scott and Carhart-Harris have proposed an experimental protocol for testing the capacity of psilocybin to increase conscious awareness in patients with DOC (52). They hypothesize psychedelics increase brain activity complexity and conscious content, in contrast to current stimulant drugs that increase arousal. However, to our knowledge there are no studies of brain complexity measures in DOC patients given stimulants. Their hypothesis relies on findings that psilocybin increases brain complexity, and that these particular measures of complexity, namely perturbational complexity index (53) and the closely related Lempel-Ziv complexity (LZC), reliably predict conscious level. They argue that psychedelic-related elevations in LZC reflect an increased richness of conscious experience, and that targeting increases in conscious content, rather than arousal, as with stimulants, may be key to increasing conscious awareness in DOC patients.

In human subjects, increases in LZC have been observed in excess of those seen in normal wakefulness with administration of psilocybin, LSD, and ketamine at psychedelic doses (54). This increase in complexity has also been demonstrated *via* other measures including electroencephalogram (EEG) and functional magnetic resonance imaging (fMRI) (55).

Psychedelics are thought to increase brain complexity primarily through 5HTRs (53). As previously discussed, 5HTR agonism is associated with increased neuroplasticity, while antagonism is associated with reduced cognitive flexibility and increased slow-wave sleep and sedation (56, 57). These receptors are most densely expressed in the cortical areas belonging to the default-mode network (DMN) (52). The DMN is known to be implicated in conscious processing as well as the subjective experience of psychedelic states (52). Strength of DMN connectivity is significantly decreased in stroke patients with DOCs, and is highly correlated with Glasgow Coma Scale scores (58). Secondarily, 5HTRs have been shown to play an important role in the control of thalamo-frontal connectivity, known to be important for consciousness (59, 60).

## Conclusions

Psychedelics may play a future role in treatment of brain injury through a variety of mechanisms. Though these are a novel class of drugs deserving close study, more data are necessary to prove their efficacy for treatment of brain injury, as historically many compounds have seemed promising *in vitro*, including likely hundreds of compounds thought to facilitate neuroplasticity and neuroprotection, but have not borne out in clinical trials (61, 62). There is already mixed evidence to suggest the use of non-classical psychedelics ketamine (63, 64), as well as tetrahydrocannabinol (THC) (65, 66) and cannabidiol (67, 68), as neuroprotectants after TBI and stroke. Presence of THC on urine drug screen is associated with decreased mortality in adult patients sustaining TBI (65). However, randomized control trials of a non-psychoactive cannabinoid analog, dexanabinol, administered once after TBI has failed to show benefit over placebo in increasing Glasgow Coma Scores at 6 months post-injury (69). One explanation for this discrepancy may be the lack of “entourage” effect whereby various cannabinoids and cannabis phytochemicals are clinically more efficacious when working synergistically compared to administration of a single cannabinoid in isolation (70), though the existence of such an effect with cannabis is contentious with mixed evidence for its existence (71). Of note, an “entourage” effect has been demonstrated with psychedelic mushrooms in which whole mushroom extracts are on the order of 10 times more potent than purified psilocin administered alone in neurobehavioral rat models (72).

Another possible explanation is that the subjective hallucinogenic effects are necessary for some, or all, of psychedelics' therapeutic effects. This remains a fundamental research question and strong evidence exists on both sides of the debate (73). Few human trials on the therapeutic effects of psychedelics at sub-behavioral “micro-” doses have been completed, but results from animal and cell studies for their use as anti-inflammatories are promising (5). In contrast, studies on the psychological effects of micro-dosing psychedelics seem to be explained by placebo effect (74, 75). Extrapolating from animal studies, the doses required at least for the anti-inflammatory effects of psychedelics are predicted to be magnitudes less than threshold for hallucinations (13). Furthermore, recent studies treating rat models of asthma with 2,5-dimethoxyphenethylamine (2C-H) which is a non-hallucinogenic 5-HT2a agonist structurally related to MDMA, suggest that psychedelics' behavioral and anti-inflammatory effects may have separate, but related, underlying mechanisms (76). A non-hallucinogenic analog of ibogaine has been shown to induce structural neuroplasticity, as well as reduce depression, alcohol-, and heroin-seeking behavior in rodents, similarly to its hallucinogenic variant (77). In contrast, Brouwer and Carhart-Harris have introduced a construct known as the “pivotal mental state” to explain the evolutionary function that 5-HT2a receptor agonism has in inducing neurologic hyper-plasticity and psychological adaptation when the body is exposed to subjective acute stress (e.g., hallucinations, intense spiritual experience, psychosis, trauma) (78). They hypothesize that “psychedelics hijack a system that has evolved to mediate rapid and deep learning” to provide a psychological “fresh start” or “rebirth.” It is likely that some, but not all, of the psychological therapeutic benefits of psychedelics is dependent on the subjective experience (73), but whether the same can be said for their use in brain injury is unclear.

Classical psychedelics have millennia of historical use, do not have significant risk of dependence, and are safe to use under close medical supervision (79, 80). Though there should be caution in over-interpreting the relevance of the aforementioned animal studies' relevance to human pathological states, this historical data should serve in effect as phase 0 and phase I studies (9). However, presumably much of this historical data is based on intermittent, infrequent dosing, so trial safety data may need to be repeated with continuous, regular doses. Further phase II trials will illuminate how these drugs may treat brain injury, particularly TBI and reperfusion injury from stroke.

Further study and design of non-psychoactive analogs may answer fundamental questions regarding the interplay of hallucinations with other properties of psychedelic therapy, as well as facilitate more practical use in acute hospital settings. The subjective hallucinogenic effects of psychedelics, with clinical psychological support, may also prove valuable in fostering recovery from brain injury from a trauma recovery rehabilitation psychology standpoint due to their anti-PTSD and anti-depression effects that are now well-documented (81, 82). Depression and PTSD significantly hinder recovery from stroke and TBI (83, 84). Psychedelics' use in this area may be more immediately clinically relevant in this area than in the neuroprotection and neuro-regeneration, where comparatively less is known. Further research is needed to determine the optimal timing after injury and route of administration for neuroprotective effects. All the compounds discussed in this paper have reasonable oral absorption. Route of administration would impact the degree and timeliness of the pharmacological effects yet there are not enough studies specifically on this aspect to make recommendations. The emerging use of intranasal route of administration may afford rapid induction into the central nervous system and could be a promising future option (85).

## Author Contributions

MK and GC contributed to conception and design of the study. MK wrote the first draft of the manuscript. MK, GC, SA, and JH wrote sections of the manuscript. All authors contributed to manuscript revision, read, and approved the submitted version.

## Conflict of Interest

The authors declare that the research was conducted in the absence of any commercial or financial relationships that could be construed as a potential conflict of interest.

## Publisher's Note

All claims expressed in this article are solely those of the authors and do not necessarily represent those of their affiliated organizations, or those of the publisher, the editors and the reviewers. Any product that may be evaluated in this article, or claim that may be made by its manufacturer, is not guaranteed or endorsed by the publisher.

## Appendix

((psychedelics) OR (psychedelic) or (psilocybin) or (psilocin) or (dimethyltryptamine) or (N,N-Dimethyltryptamine) or (N,N-DMT) or (5-MeO-DMT) or (MDMA) or (3,4-Methylenedioxymethamphetamine) or (LSD) or (lysergic acid diethylamide)) and ((disorders of consciousness) or (DOC) or (inflammation) or (stroke) or (brain injury) or (reperfusion) or (TBI) or (trauma) or (neuroplasticity)).

## Abbreviations

5HTR: 5-HT receptors
ALS: Amyotrophic lateral sclerosis
DMN: Default-mode network
DMT, N: N-Dimethyltryptamine
DOI, 2: 5-dimethoxy-4-iodoamphetamine
DOC: Disorder of consciousness
EEG: Electroencephalogram
fMRI: Functional magnetic resonance imaging
IL: Interleukin
LSD: Lysergic acid diethyl amide
LZC: Lempel-Ziv complexity
MAOI: Monoamine oxidase inhibitor
MCAO: Middle cerebral artery occlusion
MDMA, 3: 4-methylenedioxymethamphetamine
MVF: Mirror visual feedback therapy
NFAT: Nuclear factor of activated T-cells
REM: Rapid eye movement sleep
S1R: Sigma-1 receptor
TBI: Traumatic brain injury
THC: Tetrahydrocannabinol
TLR: Toll-like receptor
TNF: Tumor necrosis factor
SOD: Superoxide dismutase.
