# Erratum: Psychedelics for Brain Injury: A Mini-Review

**DOI:** 10.3389/fneur.2021.772692

**Published:** 2021-09-30

**Authors:** Frontiers Production Office

**Affiliations:** Frontiers Media SA, Lausanne, Switzerland

**Keywords:** psychedelics, neuroinflammation, neuroplasticity, stroke, brain injury, review

Due to a publisher error, the below junior reviewer was not included.

“Paolo Cardone, University of Liège, Belgium in collaboration with reviewer OG”

The publisher apologizes for this mistake. The original article has been updated.

## Publisher's Note

All claims expressed in this article are solely those of the authors and do not necessarily represent those of their affiliated organizations, or those of the publisher, the editors and the reviewers. Any product that may be evaluated in this article, or claim that may be made by its manufacturer, is not guaranteed or endorsed by the publisher.

